# Advances of tooth‐derived stem cells in neural diseases treatments and nerve tissue regeneration

**DOI:** 10.1111/cpr.12572

**Published:** 2019-02-03

**Authors:** Dianri Wang, Yuhao Wang, Weidong Tian, Jian Pan

**Affiliations:** ^1^ State Key Laboratory of Oral Disease, West China Hospital of Stomatology Sichuan University Chengdu China; ^2^ Department of Oral and Maxillofacial Surgery, National Clinical Research Center for Oral Diseases, West China Hospital of Stomatology Sichuan University Chengdu China; ^3^ National Engineering Laboratory for Oral Regenerative Medicine, West China Hospital of Stomatology Sichuan University Chengdu China

**Keywords:** central nerve system, dental stem cells, nerve repair, nervous regeneration, neural differentiation, peripheral nerve system

## Abstract

Nerous system diseases, both central and peripheral, bring an incredible burden onto patients and enormously reduce their quality of life. Currently, there are still no effective treatments to repair nerve lesions that do not have side effects. Stem cell–based therapies, especially those using dental stem cells, bring new hope to neural diseases. Dental stem cells, derived from the neural crest, have many characteristics that are similar to neural cells, indicating that they can be an ideal source of cells for neural regeneration and repair. This review summarizes the neural traits of all the dental cell types, including DPSCs, PDLCs, DFCs, APSCs and their potential applications in nervous system diseases. We have summed up the advantages of dental stem cells in neural repair, such as their neurotrophic and neuroprotective traits, easy harvest and low rejective reaction rate, among others. Taken together, dental stem cells are an ideal cell source for neural tissue regeneration and repair.

## 1 INTRODUCTION

Nervous system injuries, both central and peripheral, can lead to severe negative outcomes, including hypoesthesia and paralysis. These outcomes sharply reduce the quality of a patient's life.

Alzheimer's disease, one of the most common causes of dementia,^1^ has brought great stress to human beings. Spinal cord injuries (SCI) mainly result in the loss of sensory, motor and autonomic function^2^ and overwhelmingly depress the quality of life. For the injuries of the peripheral nervous system, facial nerve injuries, a common complication of maxillofacial surgery, usually lead to facial paralysis and affect facial expression. Injuries of the inferior alveolar nerve (IAN) and the lingual nerve, which can be complications from the extraction of impacted teeth, can cause lip numbness and impair the ability to taste.

Currently, however, there are no effective or efficient treatments for nerve injury to improve patient lives. The current curative effect is quite limited and few patients achieve complete recovery. New treatments to repair and regenerate the nervous system are in urgent need; however, their development has been a great challenge.

In the past decade, advancements in research on mesenchymal stem cells (MSCs) have made great achievements sparked by numerous reports of the application of stem cells in tissue regeneration. MSCs, first identified in the aspirates of adult bone marrow, are a group of cells that possess the ability to self‐proliferate and differentiate into multiple lineages in vitro.^3^, ^4^ It has been reported that both bone marrow‐derived stem cells (BMSCs) and adipose tissue‐derived stem cells (ADSCs) have the ability to differentiate into neuron‐like/Schwann cell‐like cells in vitro through the activation of the Notch/Wnt/SHH pathways.^5^ These cells also present a great potential for nerve repair and regeneration when transplanted into an injured sciatic nerve or the spinal cord of mice.^6^, ^7^

Recently, dental stem cells, which are derived from teeth, have come to our attention.

Different populations of cells with stem cell properties have been isolated from different parts of the tooth. These cells include dental pulp stem cells (DPSCs), periodontal ligament stem cells (PDLSCs), stem cells from human exfoliated deciduous tooth (SHED), dental follicle progenitor cells (DFPSCs), stem cells from the apical papilla (SCAP) and so on^8^. All of these cells possess the ability to self‐renewal and have multilineage differentiation in vitro. As they originate from neural crest, dental stem cells exhibit characteristics similar to neural cells, such as the high expression of neural markers and protein.

The researches of dental stem cells, one of the most crucial and critical members of the MSCS, have brought out a silver lining in the treatments of diseases using cell therapy,^9^ especially for nerve repair and regeneration. Ghazaleh et  al^9^ have summarized the usage of tooth‐derived stem cells in the treatments of diseases including myocardial infarction, acute kidney injury and others and provided a potent evidence for the application of DSCs in cell‐based treatments.

In this review, we outline the significant biological traits of the various types of dental stem cells, illustrate examples of research that present the great progress being made in nerve repair and regeneration, highlight the advantages of dental stem cells in neural repair, and sum up the neural repair abilities and mechanisms of dental stem cells. We also point out the major obstacles that need to be conquered in stem cell–based therapy for nerve injuries.

## 2 DENTAL STEM CELLS

Since the discovery of BMSCs, bone marrow has been the most utilized source of MSCs. However, the method of isolating MSCs from bone marrow is not only complicated but also destructive to donors. Therefore, an alternate source of MSCs is in demand. The first time that people considered that tooth pulp might contain MSCs was during the observation of severe tooth damage. This kind of damage penetrates both the enamel and dentin and into the pulp and stimulates a natural repair process by which new odontoblasts are formed. This process produces new dentine to repair the lesion.^8^, ^10^, ^11^ Gronthos et al demonstrated this for the first time in 2000. It is the first time when Gronthos et al^12^ isolated DPSCs from dental pulp and identified their stem cell features. Since the discovery of DPSCs, varieties of dental stem cells have been isolated and utilized, respectively, including PDLCs, SCAPs, SHEDs and DFPSCs.

**Figure 1:**
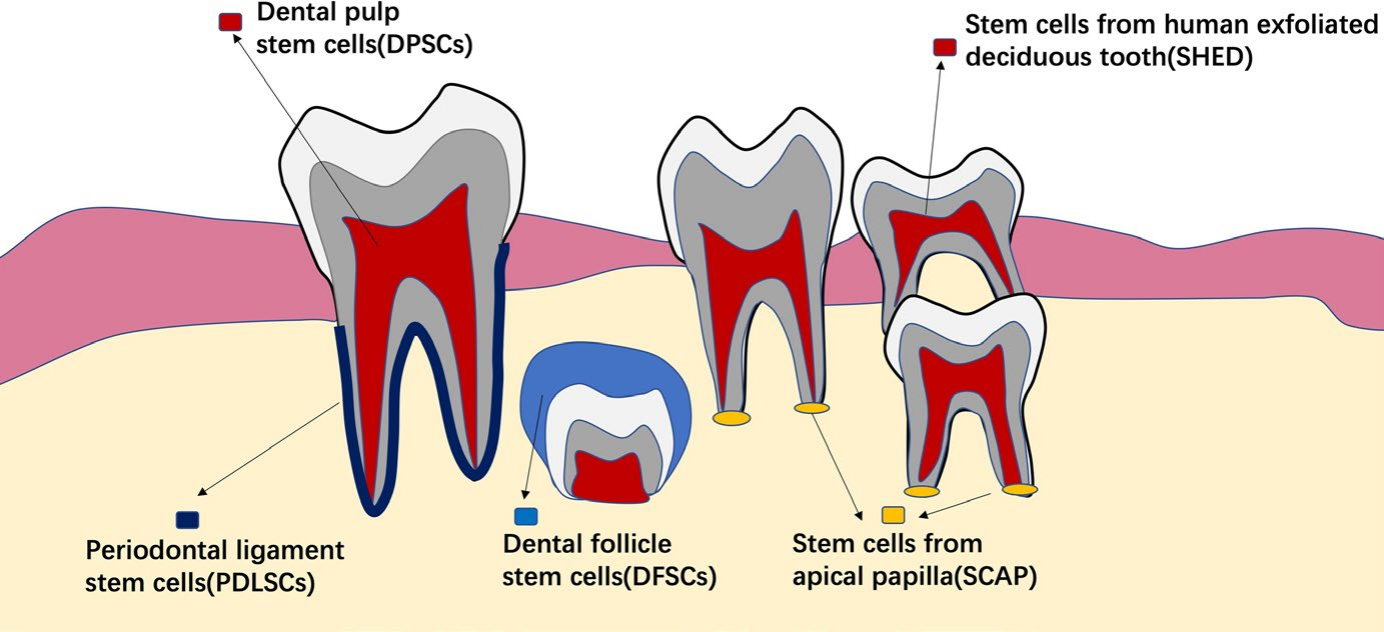
Dental stem cells, including DPSCs, PDLCs, SCAPs, SHEDs and DFPSCs, can be divided into several categories based on where they originate from. DPSCs are isolated from the dental pulp. PDLSCs are a group of cells isolated from the periodontal ligament. SHEDs are obtained from exfoliated deciduous teeth. DFSCs are isolated from the dental follicle of unerupted teeth. SCAPs are isolated from the apical papilla

Dental stem cells can be divided into several categories based on where they originate from (Figure 1). These cells share a lot of common attributes; however, they differ in certain aspects such as growth rate, gene expression and inclination for cell differentiation.^8^. However, the mechanisms that contributes to these differences remains unknown.

### 2.1 Categories of dental stem cells

#### 2.1.1 Dental stem cells include DPSCs

Dental pulp stem cells are isolated from the dental pulp. DPSCs can be classified into two main groups, including immature DPSCs and mature DPSCs. DPSCs express STRO‐1, CD146, CD105, CD73 and CD90. They also possess the capacity for multilineage differentiation. Moreover, immature dental stem cells express embryonic stem cell markers including OCT‐4, Nanog and SSEA‐3. The expression of the above markers could be maintained in subclones, indicating that DPSCs are a great source for tissue regeneration.^13^ Researches have also reported that DPSCs presented a more striking odontogenic capability than BMSCs under inducing environment.^14^ Moreover, it has been shown that dental pulp that has been diagnosed with irreversible pulpitis also contains DPSCs.^15^ These facts provide a bright future for this cellular resources.

#### 2.1.2 Periodontal ligament stem cells

Periodontal ligament stem cells are a group of cells that can be isolated from the periodontal ligament which connects the tooth root with alveolar bone. PDLSCs were firstly proposed for use in new periodontal regenerative therapies. The cell surface markers and differentiation potentiality of PDLSCs are similar to that of BMSCs and DPSCs.^16^ However, as the prevalence of periodontitis is relatively high, the ability to acquire of healthy tissue source is quite limited, and this limitation is a great challenge for the culturing of these cells.

#### 2.1.3 Stem cells from human exfoliated deciduous tooth

SHEDs, which are obtained from exfoliated deciduous teeth, have provided a potential non‐invasive source of stem cells. SHED presents relatively rapid expansion and proliferation in vitro. Furthermore, SHEDs express mesenchymal stem cell markers including STRO‐1 and CD146, CD105, CD73 and CD90.^13^ SHEDs are capable of multilineage differentiation and can differentiate into several types of cells including neural cells, adipocytes and odontoblasts. When transplanted in vivo, SHEDs can induce bone formation, generate dentin, survive in the mouse brain and express neural markers.^17^

#### 2.1.4 Dental follicle stem cells

The dental follicle is a kind of soft tissue that surrounds the developing tooth germ. The dental follicle is thought to contain stem cells that can differentiate into cementoblasts, osteoblasts and periodontal ligament cells.^18^ Similar to other dental stem cells, dental follicle stem cells (DFSCs) express similar cell surface marker and have a relatively higher proliferation rate. DFSCs were proven to possess osteogenic differentiation ability under appropriate conditions. The neural differentiation potential of DFSCs has also been shown.^19^

#### 2.1.5 Stem cells from apical papilla (SCAPs)

The apical papilla is the soft tissue that can be found at the apices of developing teeth. The dental papilla is the beginning of tooth formation and ultimately grows into dental pulp tissue.^20^ SCAPs present a rapid proliferative rate and a greater inclination for mineralization. SCAPs express typical MSC markers including STRO‐1, CD105, CD73, CD90 and CD146. As a member of the dental stem cells, SCAPs can undergo adipogenic, odontogenic and neural differentiation under inducing conditions in vitro, while SCAPs were also shown to possess the capacity for multilineage differentiation in vivo.^20^, ^21^, ^22^

### 2.2 Characteristics of dental stem cells

As a member of the MSCs, dental stem cells have the typical traits of MSCs. Like other MSCs, dental stem cells can be separated and isolated by collagenase. After approximately 1 week of culture, dental cells are able to be grown out from the dental tissue fragments.^23^, ^24^, ^25^, ^26^ Experiments have shown that most of the MSCs are fibroblast‐like and dental stem cells are as well. A large number of experiments have shown that cells that have been isolated from dental tissues, including the dental pulp, periodontal ligament and dental follicle, are fibroblast‐like and display a spindle structure.^8^ All dental stem cells have the ability to proliferate. Compared with other MSCs, such as BMSCs or ADSCs, dental stem cells display an equivalent ability to self‐renew. Among all the dental cells, DPSCs, DFCs and SHEDs exhibit higher proliferation rates and greater growth ability than others.^8^, ^23^, ^27^, ^28^, ^29^, ^30^

### 2.3 Phenotypical profile of dental stem cells

MSCs can generally be obtained from quite a few tissues including bone marrow, skeletal muscle, adipose tissue and dental pulp. These tissues commonly express a number of surface receptors including CD29, CD44, CD49a‐f, CD51, CD73, CD105, CD106, CD166 and STRO‐1 and lack expression of definitive haematopoietic lineage markers including CD11b, CD14 and CD45. Nobuyuki Kawashima et al. found that most of the dental‐derived MSCs (DMSCs) expressed typical MSC markers such as CD44, CD73, CD90 and CD105. Of the total DMSCs, 60%‐70% of cells were CD146+ dental pulp‐derived MSCs (DPMSCs).^31^ Although dental stem cells express general stem cell surface markers, no biomarkers to identify this population specifically had been discovered. Dental stem cells have been found to express STRO‐1 and CD146, which are also expressed in MSCs and BMMSCs.^12^, ^32^ However, the expression of CD146 and STRO‐1 by dental stem cells is higher than that in other MSCs such as BMSCs. Therefore, STRO‐1‐ and CD146‐positive staining has been widely used to identify dental cells. Other reports have demonstrated that there are subpopulations of DPSCs expressing STRO‐1, c‐Kit and CD34. These subpopulations possess the capability for multilineage differentiation.^33^ and have the advantages of being readily accessibility and having a higher proliferation rate. Therefore, STRO‐1, c‐Kit and CD34‐positive DSCs might be a great candidate for cell therapy. Moreover, these subpopulations have been shown to have neural repair capabilities and could contribute to peripheral nerve recovery.^34^ Although most DSCs have a similar phenotypical profile, different types of DSCs do have several distinctions among them. Phenotypical analyses showed that SHEDs have a higher expression of CD71, CD105, CD117 and CD166 compared to that of DPSCs, implying that SHEDs might be more undifferentiated.^35^

### 2.4 Differentiate ability of dental stem cells

**Figure 2:**
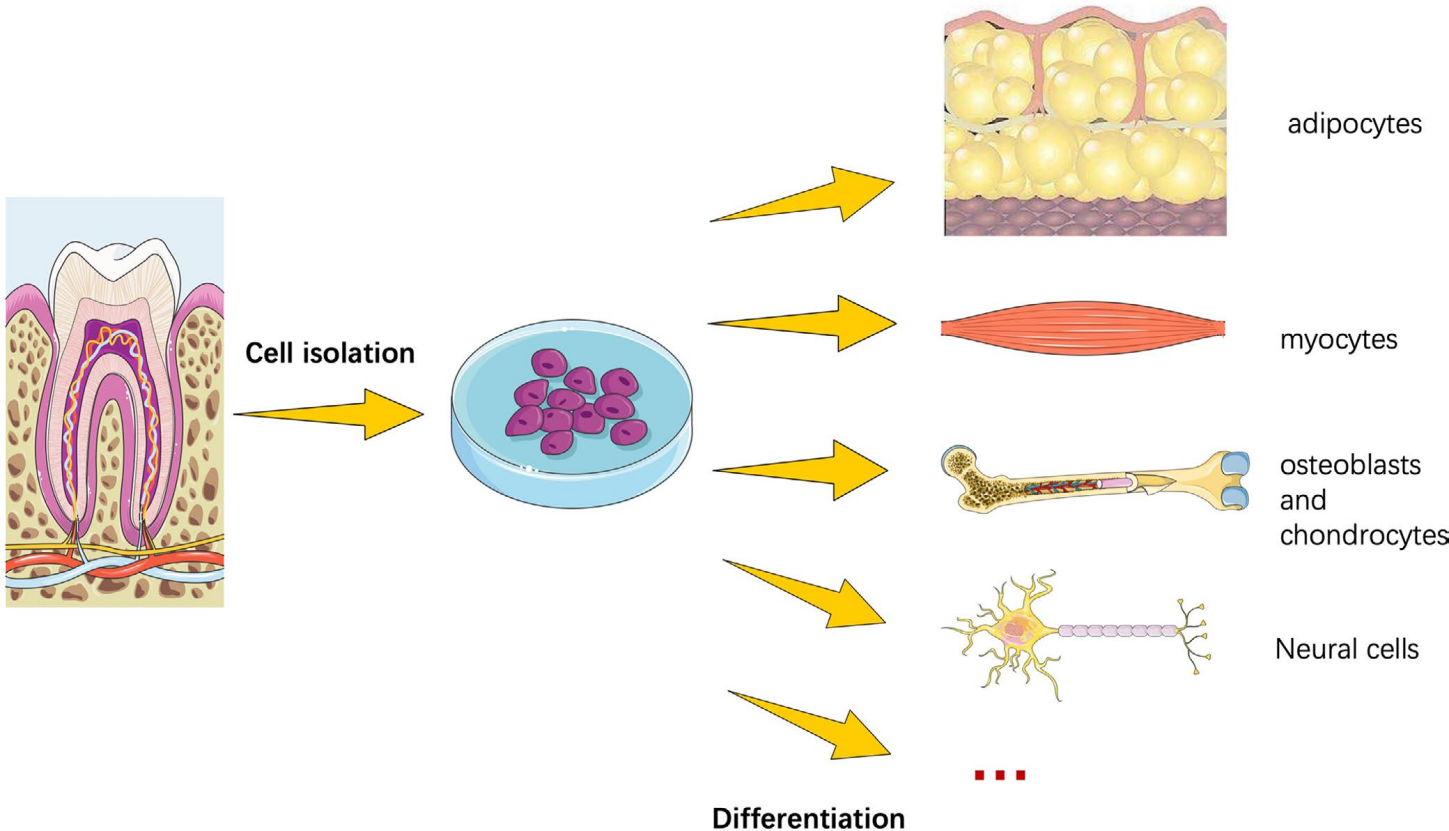
As a member of the mesenchymal stem cells, dental stem cells have the ability for multilineage differentiation abilities. Experiments have demonstrated that dental stem cells can differentiate into adipose tissues, such as adipocytes, bone tissues, including osteoblasts and chondrocytes, and nerve and neuronal tissues under suitable conditions, both in vivo and in vitro

Dental cells originate from ectodermal cells that grow around the neural tube. After migrating into the oral region, they then differentiate into cells with mesenchymal phenotypes. Therefore, they have been defined as “ectomesenchymal”.^32^, ^36^, ^37^ As a member of the MSCs, dental stem cells have the ability for multilineage differentiation abilities. It has been shown that dental stem cells possess the ability to differentiate into both endodermal, mesodermal and ectodermal tissues.^32^ Previous experiments have demonstrated that dental stem cells can differentiate into adipose tissues, such as adipocytes, bone tissues, including osteoblasts and chondrocytes, and nerve and neuronal tissues under suitable conditions, both in vivo and in vitro (Figure 2).

As the field of stem cell biology has rapidly developed, dental stem cells have become an ideal cellular sources for biological therapies and tissue engineering because of their ability to self‐renew and differentiation. DSCs are readily accessible and have less ethical constraints. All of these reasons contribute to dental stem cells being an ideal resource for regenerative medicine.

## 3 USAGE OF DENTAL STEM CELLS IN NERVOUS SYSTEM IMPAIRMENT

Dental stem cells are thought to be derived from the cranial neural crest. They can express early markers for both mesenchymal and neuroectodermal stem cells.^12^, ^17^ Considering their origin, dental stem cells inherit some typical traits of neural cells. It has been reported that dental stem cells can express specific neural markers, such as nestin, s100‐beta and GFAP. Compared to BMSCs, a type of stem cell derived from bone tissue, the percentage of neural markers expressed by dental stem cells is relatively high. Nestin‐ and GFAP‐positive cells make up only approximately 30% of BMSCs. However, those cells make up more than 90% of DMSCs.^38^ The high expression of neural markers indicates that dental stem cells can be used as an ideal seed for neural induction and regeneration. In addition to neural markers, the expression of neurotrophic factors also plays an important role. Mallappa et al found that all types of dental stem cells can express neurotrophic factors including brain‐derived neurotrophic factor (BDNF), glial cell‐derived neurotrophic factor (GDNF) and nerve grow factor (NGF). Conditioned media from dental stem cells can enhance the growth rate of Schwann cells and induce the neurite outgrowth in vitro.^39^, ^40^ In a model of rat sciatic nerve injury, dental stem cells presented neurotrophic traits and had the ability to induce axon regeneration. However, among all the dental stem cells, SCAPs have shown the greatest potential for neurotrophic effects, indicating that SCAPs could be an optimal cell source for peripheral nerve repair.^39^ Moreover, exosomes or DSCs‐conditioned medium containing exosomes is beneficial for diseases recovering.^41^ It has been reported that DPSCs exosomes possess the ability to penetrate the blood‐brain barrier and reduce or replace neuronal loss in Parkinson's disease.^41^, ^42^

In addition to the above described indirect effects on nerve repair, dental stem cells themselves can directly differentiate into neural‐like cells and participate in nerve regeneration under specific circumstances. Previous research has indicated that both DFCs and SHEDs can differentiate into neural‐like cells and possess the functions of neural cells.^43^ DPSCs, which are a significant member of dental stem cells, have been shown to differentiate towards functional neurons under appropriate microenvironments. With the contribution of epidermal growth factor (EGF), basic fibroblast growth factor (bFGF), B27 supplement and Neurobasal Media, DPSCs could form a bipolar and stellate neuron‐like morphology, which is consistent with that of functional neurons. Functional voltage‐gated Na^+^ channels are present in DPSCs when exposed to neuronal inducing conditions.^44^, ^45^ Additionally, inner neurotrophic factors such as BDNF, NT‐3 and GDNF can promote the neural differentiation of DPSCs and SHEDs. The DPSCs and SHEDs differentiated cells showed comparable functional neural activities, indicating that both adult and adolescent teeth could be considered as a cell‐based therapy source for neural diseases treatments.^46^ Moreover, it has been reported that intravenously administered DPSCs could migrate into ischaemic areas, attenuate stroke‐induced inflammation and reduce infarct volumes and cerebral oedema.^47^ Based on these above evidences, we hypothesize that DSCs could be ideal stem cell sources for central nerve repair. In conclusion, dental stem cells could be used in both peripheral and central nervous system disease treatments.

A summary of the repair mechanisms of dental stem cells can be found in Table 1. A large amount of research has built a solid foundation for nerve repair treatments in nerve repair based on dental stem cells.

**Table 1:** This table has summarized the origins, the neural differentiation abilities, the neural repair mechanisms of all types of dental stem cells, including DPSC, SHED, SCAP, DFSC and GMSCs

Type of cells	Origins	Neural differentiation ability	Disease treated	Mechanism of neural repair	References
DPSC	Dental pulp tissue	Yes	SCI	Inhibiting the apoptosis of neural cells	48
				Improving the regeneration of axons	
				Stopping the expression of axon growth inhibitors	
				Differentiating into mature neural cells	
			AD	Inhibiting the phosphorylation of Tau protein	70 ^,^ 74, 75, 76
				Increasing cell viability	
				Reducing apoptosis	
				Protecting microtubules	
			PNS	Differentiating into Schwann cell precursors	34, 89, 98, 99
				Secreting neurotrophic factors	
SHED	Pulp tissue of exfoliated deciduous tooth	Yes	SCI	Preventing demyelination and axonal loss	59, 60, 65
				Reducing TNF‐alpha	75
			AD	Providing several neuro‐reparative effects	
SCAP	Isolated from apical papilla	Yes	SCI	Reducing cell apoptosis	62
DFSC	Isolated from dental follicle	Yes	SCI	Inhibiting the expression of interleukin‐1	2
				Reducing the inflammatory response	
				Promoting neurite regeneration	
				Reducing the rate of haemorrhagic necrosis	
				Differentiating into mature neurons	
GMSCs	Gingival tissue	Yes	PNS	Differentiating into neural crest stem‐like cells	100

### 3.1 Central nervous system diseases and treatments

#### 3.1.1 Spinal cord injury

Spinal cord injury (SCI) is a severe and disabling disease resulting in the impairment of sensory and motor functions. By far, this disease does not have any effective treatments. Due to its complicated pathophysiology, which includes the loss of neurons and glia, researches leading to new treatments have faced severe challenges.^48^ The pathophysiological changes in SCI occur in two significant phases. In the acute phase, the tissue homeostasis has been broken down by the outside injurious stimulation. This kind of imbalance in the inner microenvironment induces a cascade of secondary injuries, which lead to the necrotic or apoptotic death of neurons, astrocytes and other neural cells. Furthermore, it results in irreversible damage and the loss of axons, as well as demyelination, and a stifling of the self‐repair of the spinal cord nerve.^49^, ^50^ SCI models are often applied in researches. After the exposure of vertebral column, a laminectomy was performed in order to expose the spinal cord. Moderate injury by clipping of vascular clip or a complete destroy is often used to build up SCI model. The motor and sensory recovery after SCI are often evaluated by BBB rating scale, footprint test and inclined plane test 2, 51.

In recent years, various types of stem cells, including BMSCs, ADSCs and ESCs (embryonic stem cells), have been transplanted into lesions in order to rebuild the anatomical structure of the spinal cord. Researchers have shown that stem cell–based therapy offered rats and mice a functional recovery after SCI.^52^, ^53^, ^54^, ^55^, ^56^, ^57^ However, these types of stem cells are hard to obtain. Acquirement of BMSCs or ADSCs may result in damage to bone or soft tissue in the human body. Dental stem cells, which could be isolated from an extracted tooth, are an alternative choice for stem cell therapy. Moreover, it has been proven that tooth‐derived stem cells possess great potential for SCI treatment. Kiyoshi Sakai et al found that human DPSCs could promote the recovery of rats’ spinal cord through multiple neuroregenerative mechanisms, including inhibiting the apoptosis of the neural cells of the injured spinal cord, improving the regeneration of DPSCs‐transfected axons by stopping the expression of multiple axon growth inhibitors, and differentiating into mature neural cells in order to replace the lost cells.^48^ Research has indicated that not only DPSCs but also SHEDs possess the ability to promote locomotor recovery after SCI. SHEDs can prevent demyelination and axonal loss, which helps to preserve anatomical tissue of spinal cord and contributes to SCI recovery.^58^ The neuroregenerative ability of DPSCs and SHEDs, as well as those of human BMSCs, is evaluated by the Basso Beattie Bresnahan locomotor rating scale (BBB scale). The DPSCs‐ and SHEDs‐transfected groups showed a higher BBB score, which indicated that the neural repair ability of dental stem cells was more potent than that of BMSCs. As tissue loss and cell apoptosis mainly happen during the first stage of SCI (the first 8 hours),^59^ researchers have found that transfected SHEDs could inhibit the early neuronal apoptosis. Moreover, the transplantation of SHEDs, in the acute stage of SCI, promotes an increase in the number of progenitor cells, indicating a positive effect on the lesions. Grafted SHEDs, used as a type of neuroprotective agent, downregulated the expression of GFAP, inhibited glial scar formation, delayed the decrease in S100‐beta, upregulated the expression of Kir4.1, induced tissue plasticity and finally improved the functional recovery of spinal cord contusions.^60^ Experiments also revealed that grafted SHEDs could reduce the increased level of TNF‐alpha during the early phase of SCI, through reducing the overexpression of excitatory amino acid transporter 3 (EAAT3) and of nNOS. All these functions contributed to a lower level of neuronal apoptosis.^61^ The dental apical papilla is another available source for SCI therapy. Transplantation of SCAPs to the rat SCI lesion sites improved their gait and reduced cell apoptosis.^62^ Human DFSCs have been proven to have the potential in neural repair as well. Yang et al used DPSCs, SCAPs and DFSCs in SCI repair and found that all three types, especially DFSCs, presented an inclination for nerve repair and the ability to promote function. They showed that dental stem cells could inhibit the expression of interleukin‐1 and reduce the inflammatory response to injury. Dental stem cells could promote neurite regeneration by inhibiting the expression of Ras homologue gene family member A (RhoA). Furthermore, they could reduce the expression of sulfonylurea receptor1(SUR‐1), which resulted in a decline in the rate of haemorrhagic necrosis. Moreover, DSCs could differentiate into mature neurons and promote axon growth and the nerve repair.^2^ Biomedical material combined with dental stem cells exhibits great promise in SCI treatments. Researchers have found that aligned electrospun Poly‐ε‐caprolactone/Poly‐lactide‐co‐glycolic acid (PCL/PLGA) material could be used as a scaffold for spinal cord reconstruction. DFCSs implanted onto PCL/PLGA can survive and differentiate into neural cells in SCI lesions. It is hypothesized that aligned electrospun fibres can support the spinal cord structure and induce cell neural cell differentiation.^63^ Chitosan scaffolds, a brand new biomaterial, have been shown to have an inclination for inducing dental stem cells to grow into neural cells and have a potential future use in SCI therapy.^64^ Heparin‐Poloxamer (HP), a thermosensitive hydrogel, can easily form a gel at body temperature and thus is suitable for in vivo treatments. Research has reported that HP loaded with growth factors, including bFGF and NGF, could mend nerve lesions by activating the MAPK/ERK, PI3K/Akt and JAK/STAT3 signalling pathways.^65^ HP combined with DPSCs and bFGF had a significant impact on neuronal regeneration, functional recovery and spinal cord tissue repair.^51^ Statistical analyses showed that HP‐bFGF‐DPSCs has a superior performance in nerve repair compared to HP alone or HP‐bFGF.^51^ Dental stem cell–based therapy is bringing a new hope for SCI treatment.

#### 3.1.2 Alzheimer's disease

Alzheimer's disease (AD) is an age‐related neurodegenerative disease that severely affects a person's normal daily life, with symptoms including memory loss, motor disability, linguistic disorders and cognitive deficits.^66^, ^67^ The pathological change in AD is quite complicated and includes the loss of neurons, intracellular neurofibrillary tangles and the deposition of insoluble beta‐amyloid peptides in the brain.^67^, ^68^ However, many of the pathological mechanisms of AD still remain unknown to us now. The therapeutic effect of currently available treatments for AD is still far below our expectation. For a deep investigation of AD treatments, AD mouse models have been created by overexpression amyloid precursor protein. These model mouse may develop several similar symptoms to those of AD including amyloid‐associated neuroinflammation, dystrophic neurites and synapses and so on.^69^ AD cell model is also frequently used in researches. Okadaic acid (OA)‐induced SH‐SY5Y cells present a rounder shape with retracted cytoplasm and poor adherence.^70^ Progress in the treatments of AD had been stifled for years until the usage of MSCs came to the historical stage. Several MSCs, including BMSCs and ADSCs, have been proven to be beneficial in AD recovery.^71^ It has been reported that MSCs have neural regenerative and paracrine effects, which can manipulate cell apoptosis and reduce inflammatory reactions. Experiments with animal models of AD also showed that the transplantation of MSCs could decrease the number of amyloid plaques in the brain and inhibit the secretion of inflammatory cytokines from microglia.^71^, ^72^, ^73^ All of these facts indicate that MSCs can be an alternative for AD treatment. Among the types of MSCs that can be used, dental stem cell exhibits great privilege. Wang et al showed that DPSCs could secret various growth factors to inhibit the phosphorylation of Tau protein and to promote neural stem cells proliferation. Moreover, additional experiments showed that DPSCs could restore the morphological damage, increase cell viability and reduce the levels of apoptosis in okadaic acid‐induced SH‐SY5Y cells. Furthermore, DPSCs are able to protect microtubules in neural cells. Apart from the above‐mentioned mechanisms, DPSCs possess the ability to promote neural repair in in vitro AD model.^70^ High levels of VEGF, fractalkine, RANTES, fms‐related tyrosine kinase 3 and monocyte chemotactic protein 1 secreted by DPSCs could reduce amyloid beta peptide‐induced neural degeneration and apoptosis.^74^, ^75^, ^76^ Furthermore, the DPSCs secretome is highly enriched in neurotrophic factors, amyloid beta‐degrading enzymes (NEPs) and anti‐apoptotic factors, demonstrating that DPSCs are an ideal candidate for therapy of AD.^74^ In addition to dental stem cells themselves, conditioned medium from human dental stem cells can also improve cognitive functions in mouse AD models. SHED‐CM may provide several neuro‐reparative effects that benefit the treatment of cognitive deficits and AD treatments.^75^ PDLSCs have also been shown to have the ability to reconstruct tissues destroyed by the chronic pathology of AD. The levels of apoptosis of okadaic acid‐induced SH‐SY5Y cells were lower and the expression of pTau protein decreased. These facts suggest that dental stem cells have advantages in AD treatment.

### 3.2 Peripheral nerve system diseases and treatments

Peripheral nerve injury is mainly caused by traumatic accidents or surgical complications, which may result in sensory disturbances, paralysis and locomotive disability and severely affects a person's normal daily life. Routine surgical solutions for peripheral nerve injury tend to choose the end‐to‐end/end‐to‐side neurorrhaphy in order to fix the anatomic structure of damaged nerves.

Traditional medical treatment and physicotherapeutics (massage/hot compressed on the lesion site) do not reach our expected recovery standards.^77^, ^78^, ^79^, ^80^ Autologous nerve grafting, the gold standard therapy for peripheral nerve deficits,^81^, ^82^ also has many disadvantages, including donor site paralysis, mismatching and immunological rejection.^83^, ^84^, ^85^, ^86^ Stem cell–based therapy brings new hope to peripheral nerve repair.^87^ Of all the types of MSCs, tooth‐derived stem cells present great privilege.^32^, ^36^

#### 3.2.1 Sciatic nerve injury

The sciatic nerve injury model is a classical model that is often used to study peripheral nerve injury.^6^ The construction of sciatic nerve crush model is not complicated. After exposing sciatic nerve of rats by blunt dissection, the lesion could be created by a completely cut‐off or by vascular clips compression.^88^ Once the sciatic nerve get injury, the motor function of leg would be affected. Walking track, morphological and histological analysis are often used for recovery assessment. Studies showed that DPSCs could differentiate into Schwann cells in vitro. After transplanting these differentiated DPSCs into 15‐mm rat sciatic nerve defects sites, immunohistochemistry revealed that myelinated nerve fibres and ingrowing neurites formed instead of nerve tissue lesions.^7^ Other reports also showed that STRO‐1+/c‐Kit+/CD34+ DPSCs could promote peripheral nerve regeneration and remyelination in sciatic nerve injury models by differentiating into Schwann cells precursors and secreting neurotrophic factors including NT‐3 and BDNF.^34^ Experiments also revealed that neural induced DPSCs combined with collagen scaffolds could promote axonal outgrowth and myelination. Furthermore, an upregulation of the exogenous expression of the oligodendrocyte lineage transcription factor 2 (OLIG2) gene could induce the DPSCs to differentiate into functional oligodendrocytes, which showed a therapeutic potential in sciatic nerve injury.^89^ Gingiva‐derived MSCs (GMSCs) have been shown to promote peripheral nerve repair as well by inducing remyelination by regulating the expression of the antagonistic myelination regulators, c‐Jun and krox‐20/ERG2.^90^

#### 3.2.2 Inferior alveolar nerve injury

The IAN is a branch of the mandibular nerve that belongs to the trigeminal nerve. IAN injury mainly happens during mandibular fracture or oral surgery such as the third molar extraction and results in unpleasant complications including dysesthesia, allodynia and hyperalgesia.^5^, ^91^, ^92^, ^93^, ^94^, ^95^ There have been no specific reports illustrating the usage of dental stem cells in IAN injury repair by far. However, the utilization of dental stem cells in the sciatic nerve injury model provides powerful and forceful evidences that dental stem cells may be available for IAN nerve repair.

#### 3.2.3 Facial nerve injury

The facial nerve (seventh cranial nerve) contains both motor and sensory fibres.^96^ Injury to the facial nerve results in functional and psychological problems for patients. However, the effects of medical and surgical treatments are quite poor.^97^ Stem cell–based therapy shows great advantages. Tubulation with dental pulp cells has been proved to possess the ability to cure nerve defects. Sakai et al used silicone tubes containing DPSCs to fix facial nerve lesions and proved that DPSCs could promote facial nerve regeneration both functionally and electrophysiologically.^98^ Moreover, studies have indicated that polylactic glycolic acid (PLGA) tubes filled with DPSCs could promote recovery and regeneration of injured facial nerve.^99^ In addition, GMSCs could be induced into neural crest stem‐like cells (NCSCs) in a non‐genetic way by activating the RhoA‐ROCK/YAP1 signalling pathway. The NCSCs, which were induced from GMSCs combined with a nerve conduit, had a relatively enhanced ability to facilitate the recovery of injured facial nerve, both functionally and histologically in rats.^100^

In conclusion, dental stem cells can differentiate into neural‐like/Schwann cell‐like cells to directly replace the dead neural cells and repair injury lesions. Moreover, dental stem cells could have additional neurotrophic factors that protect the nervous system and modulate immune and inflammatory reactions. Dental stem cells are a positive choice for nerve repair and regeneration.

## 4 MECHANISMS UNDERLYING DSCS‐MEDIATED NEURAL REPAIR

DSCs, as a brand new source for cell therapy, participate in the neurorestoration in several kinds of neurodegenerative disorders. The mechanisms of how they mediate repair remain complicated. However, we could conclude, as described below, that several aspects are involved, including cell replacement, paracrine effects, vasculogenesis, synaptogenesis, immunomodulation and apoptosis.^101^

First, DSCs take part in neural repair through cell replacement. In the above context, we point out that DSCs can directly differentiate into neural‐like cells, which are positive for early neural markers including nestin. Report also revealed that neural crest subpopulation which can directly differentiate into neural lineage could be isolated from dental tissue.^102^ These cells migrate to the lesion place in order to replace the necrotic tissue.^43^, ^44^, ^48^. Cell replacement also happens when transplanted DSCs recruit endogenous neural stem cells moving to the injured area.^101^, ^103^ Second, DSCs participate in neural repair by exerting paracrine effects. As we mentioned above, DSCs express neurotrophic factors including BDNF, GDNF, NGF and NT‐3. Moreover, the expression of neurotrophic factors by DSCs is highly increased when under neural inducing conditions.^40^ Conditioned media from DSCs can enhance the growth rate of Schwann cells and can induce neurite outgrowth in vitro.^39^, ^40^ Third, DSCs improve neural repair by suppressing cell apoptosis. Cell apoptosis is a major reason for neuronal loss in the AD and SCI disease models.^101^ In the SCI model, both transfected SHEDs and DPSCs could inhibit early neuronal apoptosis.^48^, ^61^ In the AD model, experiments showed that DPSCs could reduce apoptosis levels of okadaic acid‐induced SH‐SY5Y cells.^70^^,^ 74, 75, 76 Researchers have revealed that DPSCs can secrete apoptosis inhibitor proteins, including Bcl‐2, and reduced the expression of Bas,^74^ an apoptotic regulator. All these effects contribute to downregulating the response of neural cells to apoptotic signalling. DSCs help neural repair through immunomodulation. DSCs could suppress the release of TNF‐alpha, upregulate the expression of anti‐inflammatory cytokines and are ultimately responsible for promoting neural repair.^2^, ^61^, ^101^ Finally, DSCs promote vasculogenesis and synaptogenesis.^101^ Neurotrophic factors secreted by DSCs promote the regeneration of axons.^48^ Moreover, the CXCR4/SDF1‐alpha pathways, which are known to control synapse formation, were shown to be related to the transplanted DPSCs in ischaemic conditions.^104^, ^105^

In conclusion, DSCs participate in neural repair through the above six methods.

## 5 FUTURE INSIGHTS AND MAINLY OBSTACLES

A large amount of evidence has shown that stem cells, such as BMSCS and ADSCs, are available for tissue repair, including bone and muscle tissue, among others. Both clinical and in vivo experiments have provided potent evidence for the advantages of stem cell–based therapies.

Dental stem cell–based therapy has brought great hope to the tissue regeneration field, including in nerve repair. Dental stem cells possess greater promise over other sources of stem cells. First, dental stem cells are a promising and stable cell source. They are readily accessible because they can be obtained and isolated from an extracted wisdom tooth or teeth that are extracted for orthodontic purposes. Meanwhile, discarded teeth can be easily recycled from hospitals and clinics. Therefore, there are sufficient dental cell sources for stem cell therapy. Second, acquiring dental stem cells, mostly isolated from extracted human teeth, does little harm to the human body compared to BMSCs and ADSCs, which are complex to acquire and exert great damage. Third, dental stem cells acquired from human teeth do not have the possibility of inducing immunological rejection reactions. Moreover, discarded medical waste could be utilized because the various types of dental cells, including DPCSs, SCAPs, PDLSCs and DFSCs, can be isolated from a single extracted tooth. In conclusion, using dental stem cells as a cell source for stem cell–based therapies is a way to protect the environment and can successfully turn typical “waste” into “value.” Dental stem cells will become a mainstream cell source for tissue regeneration, especially neural regeneration.

Dental stem cell combined with biomaterials provides a prosperous future for nerve repairmen. The most common biomaterials that are often used and investigated in nerve regeneration and repair experiments are chitosan,^64^, ^106^ PCL/PLGA,^63^ silicone tubes,^107^ HP^51^, ^65^ and electrospun neuro‐supportive scaffolds.^108^ However, which biomaterial scaffold is the most suitable ones for neural repair remains unknown. Meanwhile, the mechanism of how dental stem cell regulates neural repair is unclear and has become a popular topic for nowadays scientific researches.

The main obstacles in the use of dental stem cells are the isolation and culture methods. The main and traditional way of culturing method is through 2D culturing. However, it has been reported that 3D culturing is a better way for cultivating for cells because it can replicate the original living environment and tridimensional living pattern of cells in vivo, as well as enhance the odontogenic differentiation ability of DPSCs.^109^ 3D culturing can restore the traits of dental stem cells in vitro to a great extent. Cell sheet technology (CST) is another popular culturing method that largely preserves the extracellular matrix and can intimate the crosstalk between cells. Dental stem cell sheets have been shown to have advantages in odontogenic differentiation.^110^, ^111^, ^112^. However, which method is the most suitable for the neural differentiation of dental stem cells is still unclear. Moreover, dental stem cells are heterogeneous and each type of dental stem cell contains several subgroups, with every subgroup having its own special traits. For example, some subgroups are inclined to undergo osteogenic differentiation, while others are inclined to undergo neural differentiation. There is still no effective way to classify these subgroups. Cell sorting techniques that can sort out the neurally inclined subgroups are needed.

As biomaterials are greatly used extensively in tissue regeneration, a 3D biomaterial scaffold, embedded with neurotrophic factors and combined with neurally inclined dental stem cell sheets may be an ideal tool for nerve repair, however, the details of this will require in‐depth research.

## 6 CONCLUSIONS

This review summarizes the traits of the various types of dental stem cells and their capacities for use in neural repair treatments. Dental stem cells are a brand new cell source for tissue regeneration. As they are derived from neural crests, their use to fix neural tissue has been recently considered. Researchers have already confirmed that dental stem cells have the capacity for multilineage differentiation, as well as the capacity to differentiate into neural‐like cells under special conditions. They can secret several neurotrophic factors, such as BDNF and GDNF, which can promote myelination and neural regeneration. Numerous in vivo experiments have provided solid evidence for the use of dental stem cells to fix central and peripheral nervous system diseases. Dental stem cells may have a great opportunity to be applied in clinical neural disease treatments in the near further. Meanwhile, dental stem cells combined with biomaterials provide a great option for neural tissue repair. Furthermore, dental stem cells have many advantages over other MSCs, including the fact that they are readily accessible and their acquisition method does less harm to the human body. In conclusion, dental stem cells have a bright future in neural tissue regeneration and repair, but there are still numerous obstacles that need to be overcome.

## CONFLICT OF INTERESTS

The authors declare no conflict of interests.
